# Exit Strategies in Natalizumab-Treated RRMS at High Risk of Progressive Multifocal Leukoencephalopathy: a Multicentre Comparison Study

**DOI:** 10.1007/s13311-021-01037-2

**Published:** 2021-04-12

**Authors:** Aurora Zanghì, Antonio Gallo, Carlo Avolio, Rocco Capuano, Matteo Lucchini, Maria Petracca, Simona Bonavita, Roberta Lanzillo, Diana Ferraro, Erica Curti, Maria Buccafusca, Graziella Callari, Stefania Barone, Giuseppe Pontillo, Gianmarco Abbadessa, Valeria Di Francescantonio, Elisabetta Signoriello, Giacomo Lus, Patrizia Sola, Franco Granella, Paola Valentino, Massimiliano Mirabella, Francesco Patti, Emanuele D’Amico

**Affiliations:** 1 Department “G.F. Ingrassia”, MS Center, Organization University of Catania, Catania, Italy; 2 MS Center I Division of Neurology, University Della Campania “L. Vanvitelli”, Naples, Italy; 3 Department of Medical and Surgical Sciences Head of Multiple Sclerosis Center Dept. of Neurosciences, University of Foggia, Foggia, Italy; 4 Fondazione Policlinico Universitario “A. Gemelli” IRCCS, Istituto di Neurologia, Università Cattolica del Sacro Cuore, Rome, Italy; 5 Università Cattolica del Sacro Cuore, Rome, Italy; 6 Dipartimento Di Scienze Mediche E Chirurgiche Avanzate, Università Della Campania Luigi Vanvitelli, Piazza Miraglia, 2, 80138 Naples, Italy; 7 Department of Neuroscience, Reproductive Sciences and Odontostomatology, University of Naples “Federico II”, Naples, Italy; 8 University of Modena and Reggio Emilia, Modena, Italy; 9 Multiple Sclerosis Centre, Department of General Medicine, Parma University Hospital, Parma, Italy; 10 Azienda Ospedaliera Universitaria “G. Martino”, Messina, Italy; 11 Institute Foundation “G. Giglio”, Cefalù, Italy; 12 Azienda Ospedaliera Universitaria “Mater Domini”, Catanzaro, Italy; 13 Department of Advanced Biomedical Sciences, University “Federico II”, Naples, Italy; 14 Department of Electrical Engineering and Information Technology, , University “Federico II”, Naples, Italy; 15 Dipartimento Di Scienze Mediche E Chirurgiche Avanzate, Università Della Campania Luigi Vanvitelli, Piazza Miraglia, 2, 80138 Napoli, Italy; 16 Department of Clinical and Experimental Medicine, Multiple Sclerosis Center, II Division of Neurology, Second University of Naples, Naples, Italy; 17 Unit of Neurosciences, Department of Medicine and Surgery, University of Parma, Parma, Italy

**Keywords:** Natalizumab, Ocrelizumab, Rituximab, Cladribine, Exit strategy, Disease activity

## Abstract

The main aim of the study is to evaluate the efficacy and safety profile of ocrelizumab (OCR), rituximab (RTX), and cladribine (CLA), employed as natalizumab (NTZ) exit strategies in relapsing–remitting multiple sclerosis (RRMS) patients at high-risk for progressive multifocal leukoencephalopathy (PML). This is a multicentre, retrospective, real-world study on consecutive RRMS patients from eleven tertiary Italian MS centres, who switched from NTZ to OCR, RTX, and CLA from January 1st, 2019, to December 31st, 2019. The primary study outcomes were the annualized relapse rate (ARR) and magnetic resonance imaging (MRI) outcome. Treatment effects were estimated by the inverse probability treatment weighting (IPTW), based on propensity-score (PS) approach. Additional endpoint included confirmed disability progression (CDP) as measured by Expanded Disability Status Scale and adverse events (AEs). Patients satisfying predefined inclusion and exclusion criteria were 120; 64 switched to OCR, 36 to RTX, and 20 to CLA. Patients from the 3 groups did not show differences for baseline characteristics, also after post hoc analysis. The IPTW PS-adjusted models revealed that patients on OCR had a lower risk for ARR than patients on CLA (ExpB_OCR_ 0.485, CI 95% 0.264–0.893,* p* = 0.020). This result was confirmed also for 12-month MRI activity (ExpB_OCR_ 0.248 CI 95% 0.065–0.948, *p* = 0.042). No differences were found in other pairwise comparisons (OCR vs RTX and RTX vs CLA) for the investigated outcomes. AEs were similar among the 3 groups. Anti-CD20 drugs were revealed to be effective and safe options as NTZ exit strategies. All investigated DMTs showed a good safety profile.

**Supplementary Information:**

The online version contains supplementary material available at 10.1007/s13311-021-01037-2.

## Introduction

Natalizumab (NTZ) has improved the possibilities to treat highly active relapsing–remitting forms of multiple sclerosis (RRMS) patients (1).

However, a long exposure to NTZ treatment in anti-JC virus (JCV)–seropositive patients expose them to a higher risk to develop progressive multifocal leukoencephalopathy (PML), a serious and potentially lethal opportunistic brain infection [2–5]. To manage PML risk, a magnetic resonance imaging (MRI) monitoring every 3–4 months has been recommended for JCV–seropositive patients on NTZ treatment for more than 18 months [5]. Furthermore, although several retrospective studies have investigated the effect of extended interval dose (EID) on reducing PML risk, the reliability of their conclusions is limited by the nonrandomized designs, and the extreme variable definitions of EID (ranging from 5 to 8 weeks) [5].

All above considered, a therapeutic switch in patients who respond to NTZ but are exposed to a high PML risk represents an important and increasingly frequent therapeutic challenge in MS clinical practice. Since highly effective drugs have been licensed for the treatment of highly active RRMS, there is an urgent need of clinical-MRI data to develop guidelines addressing/regarding exit strategies to follow in JCV-positive NTZ-treated RRMS patients at high risk of PML [6–8].

The decision to switch to another drug is always shared between neurologist and patient, and it should derive from the balance of several factors, including the risk of side effects, the maintenance of a good clinical-MRI response, and the occurrence of a clinical and radiological rebound that has been frequently observed early after NTZ discontinuation [3, 9–17].

About therapeutic options after NTZ withdrawal, the drugs targeting CD20 + B cells (rituximab (RTX) and ocrelizumab (OCR)) have proven to be very effective in suppressing inflammatory activity in RRMS [18], although not associated with a significant PML risk [19].

More recently, cladribine (CLA) tablets have been approved for highly active RRMS, but scarce data are available on the use of CLA after NTZ [7, 20].

On this background, the aim of the present multicentre real-world study was to compare the effectiveness, tolerability, and safety of a therapeutic switch to OCR, RTX, or CLA in RRMS patients treated with NTZ who were considered responder to such treatment that needed or required to stop NTZ for the high risk of PML.

## Methods

### Setting and Participants

In this retrospective observational study, we collected prospective clinical and MRI data from RRMS patients followed at 11 tertiary Italian MS centres. In detail, we identified adult RRMS patients who switched from NTZ to OCR, RTX, and CLA from January 1st, 2019, to December 31st, 2019.

Inclusion criteria were: 1) ≥ 18 years of age, 2) a diagnosis of RRMS according to McDonald revised diagnostic criteria [21]; 3) no evidence of clinical and radiological activity in the last 12 months of NTZ treatment (with a standard or EID regimen); and 4) washout period from NTZ treatment not longer than 12 weeks.

We considered for the analysis only RRMS patients who switched form NTZ for the following safety reasons:

1. a JCV index ≥ 1.5 and more than 24 NTZ infusions
2. patient’s decision to discontinue NTZ for the fear of an increased risk of PML, even if the positive JCV index was < 1.5.

The decision to stop NTZ was reached after a consultation between the neurologist and the patient, during which the risks and benefits of continuing or interrupting NTZ were clearly explained and discussed.

## Procedures and Outcomes

Patients were treated in accordance with treatment procedures and guidelines approved by European and Italian Medicines Agencies.

In detail, OCR is administered at the dosage of 600 mg/intravenous, and the first 2 infusions—each of 300 mg—are given 2 weeks apart and subsequent 600-mg infusions are given every 6 months [22].

RTX is used as off-label treatment in highly active RRMS patients [23]. It is administered as an intravenous infusion in doses of 1000 mg [24]. Subsequent doses and timing of administration are usually every 6–12 months, but no consensus guidelines exist. Among our RRMS patients, the interval between the first and second infusions was on average 7 months (range 6–9).

CLA tablets are administered in 2 treatment courses ~1 year apart [7]. The recommended cumulative dosage is 3.5 mg/kg body weight administered orally and divided into 2 yearly treatment courses (1.75 mg/kg per treatment course). Each treatment course is divided into 2 treatment cycles [7]. The first treatment course (year 1) is structured as follows: a first cycle (month 1) that starts at any given time and a second cycle (month 2) which starts 23–27 days after the last dose (~1 month after beginning first cycle).

Data were recorded retrospectively (including data until 12 months before NTZ starting, time on NTZ, and the washout period) and prospectively (until the last available visit of follow-up) from the beginning of 1 the 3 investigated drugs (the index date).

The data entry portal was iMed^©^ software's (iMed, Merck Serono SA - Geneva, Switzerland). Data were extracted on September 30th, 2020.

Disability was assessed by Expanded Disability Status Scale (EDSS) by a neurostatus-certified MS specialist. MRI data were acquired on 1.5-T scanners (the same at each centre from baseline to the end of the follow-up) and included T2- and pre- and postcontrast T1-weighted sequences [25]. Postcontrast T1-weighted sequences were acquired after intravenous injection of gadolinium contrast agent (0.1 mmol/kg). A cerebral MRI acquired within 30 days before the treatment start (during the washout period) was considered the baseline MRI, and the number of brain T2-, pre-, and postcontrast T1 lesions was recorded. Follow-up MRIs to assess disease activity were acquired at 6 and 12 months after the start of post-NTZ treatment.

## Study Endpoints

The primary study outcome was the annualized relapse rate (ARR) on investigated drugs. Additional endpoints included MRI activity after 12 months and confirmed disability progression (CDP) as measured by EDSS until the last follow-up.

Safety profile of the investigated DMTs was also investigated and reported.

A relapse was defined as the development of new symptoms or exacerbation of existing symptoms that persisted for ≥ 24 h, in the absence of concurrent illness or fever, and occurred ≥ 30 days after a previous relapse. ARR was defined as total number of relapses divided by patient–months on therapy.

CDP was defined as an increase in EDSS by ≥ 1.5 points for those with a baseline EDSS score of 0, by 1 or more points for a baseline score of ≤ 5.5, or by 0.5 points for a baseline score of > 5.5, which was sustained for 12 weeks or longer. EDSS recorded within 30 days after the onset of a relapse were excluded.

MRI activity was considered new T1-gadolinium enhancing brain lesion and/or a new or newly enlarging T2 brain lesion [26].

We defined EID if the mean interval between doses were ⩾5 weeks and standard interval dose (SID) if the mean interval between doses were < 5 weeks. The 5-week cut-off was defined a priori being the midpoint between SID (4 weeks) and EID (6–8 weeks) [27]. All patients received SID NTZ for at least 1 year, and after that, some were switched to an EID regimen due to the risk of PML.

We collected data on the safety and tolerability, reporting the frequency of Adverse Events (AEs) in accordance to EMA definition [28]. Registered AEs were severe infections requiring medication, except for uncomplicated lower urinary tract infections; AEs causing discontinuation of therapy; and AEs related to each infusion of OCR/RTX or the first dosing of CLA cycle (both reported separately).

## Protocol Approval Standard, Registrations, and Patient Consents

The study protocol was approved by the local ethics committee (Comitato Etico Catania 1 no. 140/2020/PO) of the coordinating centre (Policlinico Vittorio Emanuele, Catania, Italy), and patients provided written informed consent. The study was conducted in accordance with the ethical principles of the Declaration of Helsinki and with the appropriate national regulations.

## Statistical Analysis

All patient characteristics summary statistics are reported in terms of frequencies (%) for categorical variables, mean standard deviation (S.D.), or median with interquartile range (IQR) for continuous variables. The Kolmogorov test was used to verify data distribution. According to this latter, parametric or nonparametric test was employed. The Bonferroni test was used for post hoc analysis.

According to the Akaike information criterion, we selected the model with the best statistical inferential properties. All the models were estimated using the Breslow’s tie correction.

To consider the imbalance of the 2 groups, a propensity score (PS) was calculated as the following.

A logistic regression was performed to score all patients according to the treatment (OCR = 1 vs CLA = 0, OCR = 1 vs RTX = 0 and RTX = 1 vs CLA = 0) used as independent variable and the following covariates at baseline: age, sex, EDSS in the year prior to switch to new DMT, number of NTZ infusions, and EID during the NTZ treatment as covariates.

Inverse probability of treatment weight (IPTW) and the stabilized inverse probability of treatment weight (SIPTW) were also calculated. HRs and CI 95% were calculated.

Two generalized regression models IPTW PS-adjusted were performed to evaluate relationship between: I) ARR and treatment groups and II) MRI activity after 12 months and treatment groups. The generalized equation models employed were adapted according to the nature of variables, respectively, linear (for ARR expressed as a continuous variable) and logistic binary (for MRI activities, expressed as dichotomic).

CDP, as measured by EDSS, was compared using a contingency table.

SPSS version 21.0 was used for all analyses (IBM SPSS Statistics 21, IBM©, Armonk, NY, USA).

## Results

**Fig. 1:**
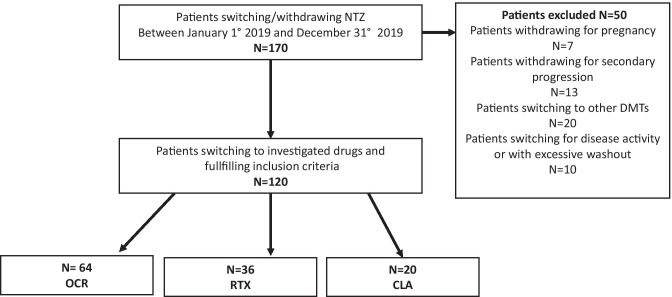
Patients’ selection flow chart. CLA = cladribine; NTZ = natalizumab; OCR = ocrelizumab; RTX = rituximab

From a total cohort of 980 RRMS patients treated with NTZ in the enrolled centres, 170 stopped NTZ for any reasons during the index window, and 120 fulfilled the required criteria. Out of them, 64 switched to OCR, 36 switched to RTX, and 20 switched to CLA (Fig. 1). Demographical and clinical characteristics of the whole cohort and groups are reported in Table 1. Overall, the entire cohort did not show differences for baseline characteristics (Table 1). After post hoc test, the results were confirmed.

**Table 1:** Baseline characteristic among the 3 groups

Variables°	OCR (64)	RTX (36)	CLA (20)	p*
Female *n*, (%)	42, (65.6)	26, (72.2)	13, (65)	ns
Age at disease onset (± mean, S.D.) (year)	24.4 ± 9.5	23.7 ± 9.9	26.5 ± 10.2	ns
Number of DMTs before NTZ	1.3 ± 1.1	1.7 ± 1.1	1.1 ± 0.8	ns
Number of relapses 12 m before NTZ start	1.6 ± 1.1	1.4 ± 1.2	1.6 ± 0.9	ns
Number of T2-weighted brain lesions 12 m before NTZ start	28.2 ± 16.1	23.8 ± 12.5	21.6 ± 13.1	ns
Number of T1-Gd + brain lesions 12 m before NTZ start	1.6 ± 2.2	1.5 ± 1.7	1.0 ± 1.2	ns
EDSS at NTZ start, median (interquartile range)	3.0 (2.0–4.5)	2.5 (2.0–4.0)	2.0 (1.0–3.0)	ns
Number of NTZ infusions	35.1 ± 26.9	40.3 ± 24.9	26.7 ± 15.8	ns
EID (*n*, %)	25 (39.1)	11 (30.6)	6 (30)	ns
EID duration (weeks)	16 ± 3.2	15 ± 4.7	14 ± 5.1	ns
Washout period (weeks)	8 ± 4.2	7 ± 3.9	6 ± 2.9	ns

Data are expressed as mean ± S.D. when otherwise specified

*DMT* disease modifying therapy; *EDSS* Expanded Disability Status Scale; *EID* extended interval dose; *NTZ* natalizumab

^*^via *χ*2, Fisher exact test or ANOVA according to the nature of variables

Table 2 shows the main clinical and radiological findings after switch among the 3 groups.

**Table 2:** Clinical and radiological findings among the 3 groups after switch

Variables°	OCR (64)	RTX (36)	CLA (20)	p*
Patients relapsing during treatment *n* (%)	5 (7.8)	5 (13.9)	4 (20)	ns
Patients with more than 1 relapse during treatment *n* (%)	0	3 (8.3)	3 (15)	0.017
Median time to first relapse median (q1–q3) (months)	3 (2–3)	6 (3–8)	3 (2.7–3.7)	ns
EDSS at baseline median (q1–q3)	3.0 (2.0–4.5)	4.0 (2.0–4.5)	2.0 (1.0–3.0)	ns
EDSS after 6 months median (q1–q3)	3.0 (2.0–4.5)	4.0 (2.0–4.5)	2.0 (1.0–4.0)	ns
EDSS after 12 months median (q1–q3)	3.0 (2.0–4.5)	3.5 (1.5–4.0)	3.0 (1.5–5.0)	ns
EDSS after 18 months median (q1–q3)	3.0 (2.0–4.5)	3.5 (1.5–4.0)	3.0 (1.5–5.0)	ns
Patients with CDP at last follow-up *n* (%)	5 (7.8)	3 (8.3)	2 (10)	ns
Patients with increased lesions load on T2-weighted or T1 Gad + weighted brain MRI lesions after 6 months *n* (%)	5 (7.8)	3 (8.3)	4 (20)	ns
Patients with increased lesions load on T2-weighted or T1 Gad + weighted brain MRI lesions after 12 months *n* (%)**	6 (9.3)	6 (16.7)	4 (20)	ns
Follow-up in months median (q1–q3)	18 (15–19)	17 (14–20)	16 (13–18)	ns

The estimated means for ARR showed a trend of significativity among the 3 groups, with value of 0.001 for patients on OCR, 0.308 for patients on RTX, and 0.500 for patients on CLA (*p* = 0.053).

The generalized regression model IPTW PS-adjusted revealed that patients on OCR had a lower risk for ARR than patients on CLA (ExpB_OCR_ 0.485 CI 95% 0.264–0.893, *p* = 0.020).

**Fig. 2:**
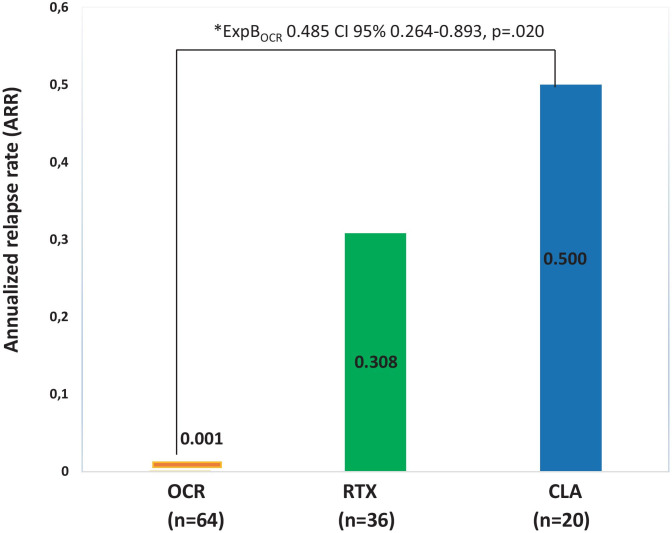
ARR endpoint (asterisk). The treatment effects were explored by a propensity-score adjustment in quintiles for age, sex, and EDSS in the year prior to switch to new DMT, number of NTZ infusions, and EID during the NTZ treatment. ARR = annualized relapse rate; CI = confidence interval; CLA = cladribine; NTZ = natalizumab; OCR = ocrelizumab; RTX = rituximab

No differences were found for the investigated outcome between OCR and RTX (ExpB_OCR_ 0.875 CI 95% 0.749–1.021, *p* = 0.089) and between RTX and CLA (ExpB_RTX_ 0.858 CI 95% 0.640–1.149 *p* = 0.304) (Fig. 2).

The generalized regression model IPTW PS-adjusted revealed that patients on OCR had a lower risk for MRI activity than patients on CLA (ExpB_OCR_ 0.248 CI 95% 0.065–0.948, *p* = 0.042).

No differences were found for the investigated outcome between OCR and RTX (ExpB_OCR_ 1.247 CI 95% 0.573–2.717, *p* = 0.578) and between RTX and CLA (ExpB_RTX_ 1.240 CI 95% 0.263–5.851 *p* = 0.786) (Fig. 2).

The CDP at the last follow-up did not differ among the 3 groups (*p* = 0.953).

No patient received a diagnosis of PML. Fourteen patients reported AEs within the first 12 months of treatment. Out of them, severe infections were reported in 3 patients on OCR, 1 on RTX, and 1 on CLA (Table 3).

**Table 3:** Adverse events among the 3 groups

	OCR (*n* = 64)	RTX (*n* = 36)	CLA (*n* = 20)
AE, within 12 months			
Patients with AEs	3 (2 urinary infections, 1 gastrointestinal infection)	1 (urinary infection)	1 (1 genitourinary infection)
First-dosing AEs			
Patients with first-dosing AEs	5 (headache, flushing, articular pain)	3 (headache, flushing, articular pain)	1 (seborrheic dermatitis of the scalp)
AEs resulting in DMT discontinuation, within 12 months			
Patients discontinuing for safety concerns	0	0	0

*AEs* adverse events; *OCR* ocrelizumab; *RTX* rituximab; *CLA* cladribine; *DMT* disease modifying therapy

First-dosing AEs were reported in 5 on OCR and in 3 patients on RTX. One patient on CLA after the first cycle reported seborrheic dermatitis of the scalp. None of the AEs reported lead to DMT discontinuation.

## Discussion

Our study revealed a lower risk of experiencing relapses and new MRI activity for patients that switched from NTZ to OCR than CLA. Contrariwise, no differences were found between those switching to RTX and CLA.

Regarding CDP, no differences were found, at the end of the follow-up, between the 3-switching group.

Overall, all DMTs revealed a good safety profile with no cases of PML.

The increased risk of PML in NTZ long-treated patients who show JCV antibodies positivity represents a matter of great concern in clinical practice. Different schemes of NTZ monitoring and/or administration, as EID regimen, have been proposed and evaluated, but they do not cancel PML risk and its consequences [5, 29, 30].

We could speculate that our results reflect the different mechanisms of action and pharmacodynamics of the investigated DMTs.

Little is known about the reasons of clinical and radiological rebound after NTZ discontinuation. It was considered the role of increased percentage of activated T cells producing cytokines in the peripheral circulation during NTZ treatment [31].

Real-world observational data about OCR as NTZ exit strategy are recent, whilst efficacy and safety of RTX have been highlighted since 2016 [9, 32–35].

A recent study investigated 42 RRMS patients who switched to OCR from NTZ after EID (5–8 weeks) and who were followed up for 6 months, clinical relapses occurred during the first 3 months of observation in 5 patients, and the EDSS remained stable in 38 (90%) patients. No serious AEs were described [34].

The most relevant observational study compared patients switching from NTZ to RTX (*n* = 114) to patients switching to fingolimod (*n* = 142) with an average follow-up of 1.5 years. Here, relapses occurred in 1.8% of RTX-treated patients compared with 17.6% of those who switched to fingolimod. The rates of AEs (5.3% vs 21.1%) and treatment discontinuation (1.8% vs 28.2%) were also lower in RTX groups. These results have been confirmed by a number of recent case series [19, 32, 33].

In our cohort, OCR was associated to lower relapse and lower MRI activity than CLA.

The comparative efficacy of CLA versus other DMTs in naïve patients has been analysed through meta-regression and matching-adjusted indirect treatment comparison approaches [36]. In detail, for the outcome ARR, CLA tablets were predicted to be less efficacious than OCR (relative risk 1.06, CI 95% 0.78–1.45) [36].

CLA is considered an immune reconstitution therapy (IRT) [37, 38]. Characteristics of IRTs include transient reductions of B and T lymphocyte counts and/or select lymphocyte subtypes, followed by a recovery period in which the B and T populations gradually recover, and immune function is restored. The reconstituted lymphocyte population usually begins within weeks after each treatment course in the first and second years and stabilized on return to baseline [39–42]. Such mechanism of action could explain why the first relapse in CLA group happened between the first and second trimesters from the therapeutic switch. Such timing could coincide to partial reconstitution of different T cells subtypes as pooled data from clinical trials showed [38, 42–45].

A previous short report by Mohon et al. [20] analysed 17 patients switching from NTZ to CLA with a median follow-up of 9.7 months (range 1.5–15 months) [20]. No patients presented a clinical relapse during the observation period, and only 2 patients showed new T2 lesions on brain MRI. [20]. However, the absence of comparisons or inferential statistical models represent limits of this study.

Our observational study firstly compared 3 high-efficacy DMTs employing a generalized model IPTW PS-adjusted for baseline characteristics to mitigate unbalance among groups, and this latter certainly constitutes an element of strength of the study.

Although IPTW has not been deeply evaluated in the context of small sample sizes, simulation studies revealed that, even in case of small study samples or low prevalence of treatment, both neighbour matching and IPTW PS can yield unbiased estimations of treatment effect [46, 47].

However, our study has some limits.

As this is a retrospective study, not all participants have the same follow-up, and the characteristics of sample size warrant cautious interpretation of the data. Moreover, we did not report lymphocytic count from investigated patients, and it could have added further data about the type and timing of lymphocytic subset repopulation.

In conclusion, prospective/longitudinal studies are needed to better clarify if switching to OCR is the choice with the best risk/benefit ratio as exit strategy after NTZ interruption because of unacceptable high risk of PML.

## Supplementary Information

Below is the link to the electronic supplementary material.

Supplementary file1 (PDF 1900 KB)

Supplementary file2 (PDF 1900 KB)

Supplementary file3 (PDF 1900 KB)

Supplementary file4 (PDF 1900 KB)

Supplementary file5 (PDF 1900 KB)

Supplementary file6 (PDF 1900 KB)

Supplementary file7 (PDF 1900 KB)

Supplementary file8 (PDF 1900 KB)

Supplementary file9 (PDF 1900 KB)

Supplementary file10 (PDF 1900 KB)

Supplementary file11 (PDF 1900 KB)

Supplementary file12 (PDF 1900 KB)

Supplementary file13 (PDF 1900 KB)

Supplementary file14 (PDF 1900 KB)

Supplementary file15 (PDF 1900 KB)

Supplementary file16 (PDF 1900 KB)

Supplementary file17 (PDF 1900 KB)

Supplementary file18 (PDF 1900 KB)

Supplementary file19 (PDF 1900 KB)

Supplementary file20 (PDF 1900 KB)

Supplementary file21 (PDF 1900 KB)

Supplementary file22 (PDF 1900 KB)

Supplementary file23 (PDF 1900 KB)

Supplementary file24 (PDF 1900 KB)

## Declarations

### Protocol Approval Standard, Registrations, and Patient Consents

The study protocol was approved by the local ethics committee (Comitato Etico Catania 1 no. 140/2020/PO) of the coordinating centre (Policlinico Vittorio Emanuele, Catania, Italy), and patients provided written informed consent. The study was conducted in accordance with the ethical principles of the Declaration of Helsinki and with the appropriate national regulations.
